# Case of Puerperal Convulsions, in Which Neuralgic Symptoms Were Singularly Exhibited

**Published:** 1856-08

**Authors:** James E. Reeves

**Affiliations:** Philippi, Va.


### BUFFALO MEDICAL JOURNAL

AND

### MONTHLY REVIEW.

**VOL. 12.** AUGUST, 1856. **NO. 3.**

### ORIGINAL COMMUNICATIONS.

ART. I.—*Case of Puerperal Convulsions, in which Neuralgic Symptoms were singularly exhibited.* By James E. Reeves, M. D., Philippi, Va.

Dr. Hunt: The following case came under my observation and treatment, and if you think it of sufficient importance, you are at liberty to publish it in the pages of your Journal, and oblige,

Yours,

James E. Reeves.

Philippi, Va., June 15, 1856.

Feb. 1st, 2 o'clock, A. M. A messenger came in great haste, and desired me to visit Mrs. T., who, he said, had been complaining of headache, &c., which suddenly resulted in a violent convulsive paroxysm.

I arrived at her dwelling 2, A. M., and found her just recovering from a second convulsion; her face was enormously swollen, of a dark-red violet color; respiration loud and hissing; pupils dilated and fixed; pulse 120, and weak. In half an hour longer she sufficiently recovered consciousness to recognize those around her, and inquired what had happened "that they had sent for the *Doctor?*"

Her history is as follows: Age 36; of medium height; short neck; dark hair and eyes; of good health; the mother of four children, the youngest 5 years old; became pregnant for the fifth time somewhere about the last of August, 1855.

At a very early period of her pregnancy, the peculiar reflex irritation of the stomach set in, and "morning sickness," as it is called, came on with unusual severity, so much so that she was completely incapacitated from attending the domestic affairs of her family; and by the fourth month she suffered to such a degree of helplessness that she was compelled to keep her bed the greater portion of her time.

During the latter part of the fifth month, however, the sickness of the stomach considerably diminished, but only to be replaced by other and more alarming symptoms: she grew full and plethoric, with which was united headache, occasional perverted vision, flushing of the face, vertigo, confusion of mind, and extreme venous distention in the lower extremities. In this state she began to despond, believing her *unusual* suffering condition to be the sure precursor of death in her confinement; her former lively countenance gave place to an expression of the deepest gloom. As she further advanced, all the symptoms gradually grew more and more distressing, when on yesterday, after a sudden increase of headache—constant vertigo; throbbing of the temples; confusion of mind; a sensation of sparks of fire dancing before her eyes; all which, this morning, resulted in the convulsive paroxysm I was summoned to witness.

She is now, according to the estimation of her friends, in her six and a-half month.

Without paying attention to her inquiry, as to the cause of my visit, I tied up her arm and took blood to the amount of twenty-four ounces; after which the pulse became soft, and reduced to 90; the turgidity of the face was considerably lessened, and a marked improvement of the mind was apparent. As she had not had a free motion from the bowels during the twenty-four hours previous, I ordered a brisk cathartic dose, and cold applications to the head. To be kept up until headache was relieved.

I remained with her eight hours, during which time the physic operated, and she felt much relieved. Before leaving for home, I cautioned the husband to have her pay strict attention to the state of her bowels, give salts or castor oil if necessary, and should the symptoms again appear premonitory of convulsions, to have her freely bled, and cold water unsparingly used upon upon the head and shoulders.

Feb. 14th, 8 o'clock, A. M. Sent for again, to see Mrs. T.; arrived 9,

A. M., and had the pain of witnessing her suffer the most violent convulsion I had ever witnessed. Yesterday morning she suffered from increased headache; giddiness; ringing noise in the ears; obscure vision, with sickness at the stomach and vomiting, for which she was bled—yesterday, 10 o'clock, A. M.—but without affording her any apparent benefit. At 1 o'clock this morning she was violently convulsed; at half past 3 she suffered another; at 6, another; and at 9, another, attended with stupor, lividity of the countenance and apparent suffocation.

I drew blood again, to the amount of twenty ounces, and used unsparingly the cold douche to the head and shoulders. In a few minutes after, she was calm, the pulse reduced in tone, 100. At half past 12 she was again convulsed, followed by another at 1 o'clock; and another, and another, every twenty or thirty minutes, throughout the evening; pulse very weak and frequent; the extremities cold.

On examination the os uteri, it was felt slightly dilated and patulous, but could not detect the least expulsive efforts going on in the womb. At 6, P. M., her condition appeared hopeless—the paroxysms continuing; pulse almost imperceptible. I concluded, but not without much hesitation, to rupture the membranes, which I accordingly did at 7, P. M. In half an hour, reäction had taken place, the skin felt warm, and brisk uterine action ensued. She became furious, and it was with the greatest difficulty that the friends could keep her in bed; she imagined that she was from home, and begged earnestly to be permitted to return. At 9, P. M., the head began to press heavily upon the perineum; a little longer, and the head passed out the vulva, immediately followed by the shoulders, and after much painful anxiety, I had the satisfaction of looking upon a living male child, of seven months, weighing about four pounds.

In ten minutes or so, after the birth of the child, the placenta came away, and she fell into a deep stupor, with now and then twitching of the tendons; pupils dilated; loud respiration; pulse 120.

Applied hot bricks to the feet, and a large blister to the nape of the neck and shoulders. Ordered strips of cotton dipped in cold water, and kept constantly upon the head.

Fourteen hours after delivery. She has just awoke as from a sound sleep, rational, but entirely ignorant of her previous condition; does not know that she is the mother of another child; saw the child, and inquired whose it was. Pulse 130, and somewhat tense; face flushed; thirst; still complains of headache.

*Administered* hyd. sub. mur. x grs.; rhei pulv. v grs.; morph. sulphas, ½ gr.

Ordered the cold strips kept to the head constantly.

Feb. 15th, twenty-four hours after delivery. Mind clear, and no return of convulsions; calomel has operated freely, and bladder emptied without difficulty; pulse 115, compressible; has slept soundly for four hours; complains of her neck being very sore from the blister.

Ordered ½ gr. morphia to be taken at 8 o'clock to-night.

Feb. 16th, 7, A. M. Has not slept very well during the night; complains of great muscular soreness; headache; pulse 130; tongue coated; urgent thirst.

℞ Tart. antimonii, gr. v.

Morphiæ sulph., gr. iv.

Aquæ, ℥vi.

Spts. nitr. dulc., ʒij.

Dose: a tablespoonful every hour or two.

Feb. 17th. Did not sleep well last night; complains of violent pain in the *dorsum* of the wrists, cannot move the hands without suffering intense pain; pulse 120, and small; pain in the head not so severe; tongue coated; urgent thirst; condition of the bowels good; thinks she could relish a little food.

Ordered the antimonial solution continued, and an opiate embrocation applied freely to the wrists.

Feb. 20th. Has suffered most excruciatingly from pain of the wrists—it has assumed a periodical form; there is an afternoon exacerbation daily; not much headache; pulse 120; thirst not so urgent; a little increased desire for food; mind unclouded; says if it were not for the severe pain in the wrists she should feel quite well.

Advised ½ gr. morphia to be taken at nights, at bed-time, to procure rest, and sulph. quinine to be taken in xx gr. doses daily, an hour or two before the evening exacerbation.

Feb. 23d, 4 o'clock, P. M. Had the pleasure of finding my patient sitting up in bed, *nursing the baby;* yesterday the pain in the wrists was not so severe, usually came on about 1, P. M.; this evening is free from pain; pulse 100; no headache; appetite good.

Ordered 10 grs. of quinia for to-morrow morning.

Feb. 27th. Has not had pain in the wrists since the evening of the 23d, then it was not so severe, and came on about 6½, P. M.

Her countenance has assumed its natural lively expression. Is able to be out of bed an hour or two at a time. Has not suffered from pain of the wrists for several days. Cautioned the nurse as to her diet, and left her, thinking she would not need any further attention.

*Six weeks later*, April 9th. I was very much surprised to learn from her husband this morning, that she suffered from pain in the wrists regularly every two weeks. But, with this exception, she is very well able to be about out of doors.

Advised 20 grs. quinia to be taken a few hours before the anticipated pain; her bowels to be kept regularly open with rhubarb and magnesia.

April 24th. Had the pleasure of seeing her. She was looking very well. Yesterday was the usual day of pain; did not suffer so severely as heretofore.

Ordered the quinia in a 25 gr. dose before the next period.

Her babe had grown finely.

May 27th. She is entirely well; has not suffered an attack of pain in four weeks.

Child very healthy.

*Remarks.* The reader will observe that this patient enjoyed a period of two weeks of comparative ease, between the first attack of convulsions and the last, when the membranes were punctured and the birth successfully accomplished; that she had no attack of convulsions subsequent to delivery, but, during convalescence, suffered pain in the wrists, with a marked exacerbation every two weeks: exactly corresponding with the length of the interval between the first and last seizure of the convulsive paroxysms.

I believe if delivery had not been brought about at the time it was, this case must have proved fatal.

